# Mechanism for Improved Curie Temperature and Magnetic Entropy Change in Sm-Doped Fe_88_Zr_8_B_4_ Amorphous Alloys

**DOI:** 10.3390/ma16237274

**Published:** 2023-11-22

**Authors:** Zhe-Rui Zhang, Xiang-Jie Liu, He-Teng Zhang, Qiang Wang, Ding Ding, Ben-Zhen Tang, Peng Yu, Jin-Lei Yao, Lei Xia

**Affiliations:** 1 Institute of Materials & Laboratory for Microstructure, Shanghai University, Shanghai 200072, China; zhangzherui@shu.edu.cn (Z.-R.Z.); liuxiangjie@shu.edu.cn (X.-J.L.); 22722611@shu.edu.cn (H.-T.Z.); mat_wq@shu.edu.cn (Q.W.); d.ding@shu.edu.cn (D.D.); 2 Chongqing Key Laboratory of Photo-Electric Functional Materials, College of Physics and Electronic Engineering, Chongqing Normal University, Chongqing 401331, China; bz.tang@cqnu.edu.cn (B.-Z.T.); pengyu@cqnu.edu.cn (P.Y.); 3 Jiangsu Key Laboratory of Micro and Nano Heat Fluid Flow Technology and Energy Application, School of Physical Science and Technology, Suzhou University of Science and Technology, Suzhou 215009, China; jlyao@usts.edu.cn

**Keywords:** amorphous alloy, glass formation ability, Curie temperature, magnetocaloric effect, magnetic entropy change

## Abstract

In the present work, Fe_88_Zr_8−x_Sm_x_B_4_ (x = 2, 4) amorphous alloys (AAs) were successfully synthesized into the shape of 40-micrometer-thick ribbons and their magnetic properties were measured. The Fe_88_Zr_8−x_Sm_x_B_4_ (x = 2, 4) AAs exhibited a rather high maximum magnetic entropy change (−Δ*S_m_^peak^*): ~3.53 J/(K × kg) near 317 K for x = 2 and ~3.79 J/(K × kg) near 348 K for x = 4 under 5 T. The effects of a Sm substitution for Zr on the Curie temperature (*T_c_*) and −Δ*S_m_^peak^* were studied and compared to those of Nd and Pr substitutions, for the purpose of revealing the mechanism involved in more detail.

## 1. Introduction

With global warming and the increasing population, the demand for air conditioners has increased dramatically in recent years. Conventional vapor compression/expansion refrigeration technology, which is currently the major technology for room-temperature refrigeration, has several disadvantages, such as a low efficiency and environmental unfriendliness. For example, the energy utilization efficiency of traditional refrigeration technology is generally less than 10%, and its refrigerant (freon) is harmful to the ozone layer and leads to holes in the ozone layer, which, in turn, exacerbates global warming [1,2,3,4]. Therefore, it is vitally urgent to improve refrigeration with a novel solution that is not harmful to the environment and has a higher efficiency.

Magnetic refrigeration (MR) technology, using magnetocaloric materials as solid refrigerants, avoids most of the disadvantages of traditional refrigeration technology. For instance, the efficiency of MR technology is much higher and refrigerators that use MR technology are more compact due to the solid refrigerant [3,4,5,6,7]. In addition, MR technology is friendly to the environment because it is free of ozone-depleting gas [6,7]. The application perspective of MR technology depends mainly on the magnetocaloric property of the magnetic refrigerant. The magnetocaloric property, namely the magnetocaloric effect (MCE), is an intrinsic property of magnetic materials that was first observed in iron by E. Warburg in 1881 [8]. The magnetic entropy of a magnet decreases with the ordering of magnetic moments upon magnetization, and increases with demagnetization. The decrease/increase in magnetic entropy under adiabatic conditions leads to the heating/cooling of the magnet, which can be applied to achieve refrigeration.

Magnetocaloric materials can generally be sorted into two categories according to the magnetic phase transition (MPT) that they experience near their Curie temperature (*T_c_*), or the shape of their magnetic entropy change (−Δ*S_m_*) curves. Materials undergoing a first-order MPT show a sharp −Δ*S_m_* curve, which results in an ultra-high maximum −Δ*S_m_* (−Δ*S_m_^peak^*), but a narrow working temperature range and a low refrigeration capacity (*RC*) [9,10,11,12,13]. In contrast, second-order MPT materials show a broadened −Δ*S_m_* curve that brings about a rather high *RC* and a wide working temperature range, both of which make second-order MPT materials more suitable to be used as refrigerants in magnetic refrigeration appliances, because a broadened −Δ*S_m_* profile is vital for an Ericsson cycle [14,15,16,17,18,19]. As one of the important categories of second-order MPT materials, amorphous alloys (AAs) show unique characteristics that make them more suitable as magnetic refrigerants, such as formability within a wide compositional range and a tunable *T_c_* as a result, better mechanical properties and corrosion resistance than their crystalline counterparts, low eddy current loss due to their high electric resistance, low hysteresis loss due to their excellent soft magnetic properties, and so on [20,21,22]. Amorphous magnetocaloric alloys are generally divided into two groups: rare-earth (RE)-based and transition-metal (TM)-based AAs. RE-based AAs, especially Gd-based AAs, show excellent glass formability (GFA) and outstanding magnetocaloric properties with a rather high −Δ*S_m_^peak^* at low temperatures [17,19,23,24]. However, the GFA and −Δ*S_m_^peak^* of these AAs decrease dramatically with their increasing *T_c_*, and thus, Gd-based AAs with a *T_c_* near room temperature exhibit a very low −Δ*S_m_^peak^* and a poor GFA, which makes them hard to fabricate [25,26,27]. In contrast, TM-based AAs, represented by Fe-based AAs, exhibit a fairly good GFA and can be easily prepared within a wide compositional range, which makes their *T_c_* tailorable within the operating temperature interval of a domestic refrigeration appliance [28,29,30,31,32,33,34,35]. The shortcoming of Fe-based AAs is their low −Δ*S_m_^peak^*; for example, the −Δ*S_m_^peak^* under 5 T of Fe-Zr-B ternary glassy alloys is usually not higher than 3.3 J/(K × kg) near room temperature [28,29]. As a consequence, one of the key issues for AAs applied industrially in MR is how to improve the −Δ*S_m_^peak^* to make it as high as possible near room temperature.

It is well known that microalloying is a useful way to achieve a better GFA and physical properties of AAs. In our preliminary works, we added a small amount of various elements and successfully improved the magnetocaloric performance of the Fe-Zr-B ternary AAs [31,32]. For instance, the −Δ*S_m_^peak^* under 5 T of Fe_88_Zr_8_B_4_ AAs was enhanced to ~3.42 J/(K × kg) by a minor Co addition and was further improved to ~3.55 J/(K × kg) by a minor Nb addition [31]. A minor Sm substitution for the Fe of Fe_88_Zr_8_B_4_ AAs led to an enhanced −Δ*S_m_^peak^* up to 3.55 J/(K × kg) [31]. However, microalloying by the substitution of other elements for Fe is finite for the improvement in the −Δ*S_m_^peak^*.

More recently, we found that the −Δ*S_m_^peak^* of Fe-Zr-B ternary AAs was further increased by a minor substitution of RE elements for the Zr, and a larger −Δ*S_m_^peak^* was achieved in the Fe_87_Ce_8_B_5_ AA ribbon (3.65 J/(K × kg) at 282.5 K) [33], Fe_88_Zr_4_Pr_4_B_4_ glassy ribbon (4.0 J/(K × kg) at 323 K) [34], and Fe_88_Zr_4_Nd_4_B_4_ glassy ribbon (4.1 J/(K × kg) at 335 K) [35]. The Sm element, with similar characteristics to Pr and Nd, also seemed to be a good substitute for Zr in improving the magnetocaloric performance of Fe-Zr-B AAs. Additionally, the mechanism for the effect of various kinds of RE substitutions on the magnetic and magnetocaloric performance has not been systematically investigated, and an understanding of the magnetocaloric performance of Fe-based AAs with various RE elements is helpful for developing better Fe-based magnetocaloric AAs with a high −Δ*S_m_^peak^* within the operating temperature interval of a domestic magnetic refrigerator. In the present work, we studied the effect of a Sm substitution for Zr on the GFA and the magnetic and magnetocaloric performance of Fe_88_Zr_8_B_4_ AAs. Based on the experimental results obtained in this work and our previous works, the mechanism for the effect of an RE substitution on the *T_c_* and −Δ*S_m_^peak^* of an Fe_88_Zr_8_B_4_ basic alloy was investigated in more details.

## 2. Experimental Procedures

By arc-melting Fe, Zr, and Sm metal blocks (purity > 99.9 at.%) and an Fe-B pre-alloy at least four times, ingots with nominal compositions of Fe_88_Zr_8−x_Sm_x_B_4_ (x = 2, 4) were synthesized in a high-vacuum furnace protected by an Ar atmosphere. Due to the evaporation amount of the Sm element accounting for almost 4.39 wt.% of the total mass of the ingot during the arc-melting process, the actual concentration of Sm in the alloy ingots was estimated to be 1.91 at.% when x = 2 and 3.83 at.% when x = 4. Considering that there was not a significant deviation in the Sm concentration, the nominal composition of the two alloys is still used in the following text. The Fe_88_Zr_8−x_Sm_x_B_4_ (x = 2, 4) ribbons were prepared using a melt-spinning technology at a rotation rate of ~55 m/s. The distance from the nozzle to the wheel was about 2 mm and the melts were ejected onto the wheel surface under an ejection pressure of 0.5 MPa when the alloy melts were flowable. The Fe_88_Zr_8−x_Sm_x_B_4_ (x = 2, 4) as-quenched ribbons with an average thickness of approximately 40 μm were picked for subsequent investigations. The amorphous characteristics of the samples were firstly examined with a PANalytical X-ray diffractometer (XRD, Panalytical, Netherlands) using Cu *K_α_* radiation, and further ascertained using the differential scanning calorimetry (DSC) curves measured using a NETZSCH 404 C calorimeter (Netzsch, Selb, Germany). The thermal properties of the AAs were obtained from the DSC curves measured at a heating rate of 0.333 K/s. A microstructural observation of the Fe_88_Zr_6_Sm_2_B_4_ amorphous ribbon was performed using a JEOL JEM-F200 cold field emission high-resolution electron microscope (HREM, JEOL, Tokyo, Japan). The HREM sample was polished using a GATAN 691 precision ion-polishing system (Ametek, Berwyn, PA, USA). A vibrating sample magnetometer (VSM) module on a Quantum Design model 6000 physical properties measurement system (PPMS, Quantum Design, San Diego, CA, USA) was employed for the magnetic measurements of the ribbons.

## 3. Results

### 3.1. The Structure of the Fe_88_Zr_8−x_Sm_x_B_4_ (x = 0, 2, 4) as-Quenched Ribbons

**Figure 1:**
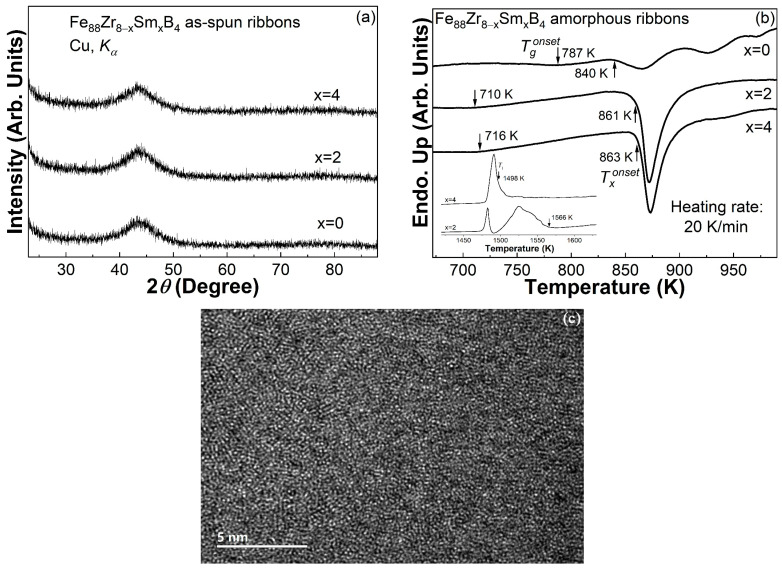
(**a**) The XRD patterns of the Fe_88_Zr_8−x_Sm_x_B_4_ (x = 0, 2, 4) as-spun ribbons; (**b**) the DSC curves of the Fe_88_Zr_8−x_Sm_x_B_4_ (x = 0, 2, 4) amorphous ribbons, with the melting behaviors in the inset; and (**c**) the HREM image of the Fe_88_Zr_6_Sm_2_B_4_ amorphous ribbon.

The XRD patterns of the Fe_88_Zr_8−x_Sm_x_B_4_ (x = 0, 2, 4) as-quenched ribbons are illustrated in Figure 1a. The smooth amorphous diffraction peak near 43 degrees and the invisible crystalline peaks suggested that these ribbons were amorphous. The sharp exothermic crystallization peak followed by the smooth endothermic glass transition hump in these DSC traces of ribbons, as shown in Figure 1b, also ascertained their amorphous characteristics.

### 3.2. Thermal Properties and GFA of the Fe_88_Zr_8−x_Sm_x_B_4_ (x = 0, 2, 4) AAs

The thermal properties of the Fe_88_Zr_8−x_Sm_x_B_4_ (x = 2, 4) AAs, including the onset temperature of the glass transition (*T_g_^onset^*) and primary crystallization (*T_x_^onset^*), were identified, as labeled on the three DSC traces of ribbons in Figure 1b. For comparison, the DSC trace of the Fe_88_Zr_8_B_4_ basic alloy is also shown in Figure 1b. The addition of Sm obviously enlarged the supercooled liquid region (∆*T_x_* = *T_x_^onset^* − *T_g_^onset^*) [36] of the Fe_88_Zr_8_B_4_ amorphous alloy, which indicated an increased thermal stability against crystallization. The large ∆*T_x_* of ~150 K for the Fe_88_Zr_8−x_Sm_x_B_4_ (x = 2, 4) AAs was much higher than those of the other Fe-Zr-B-based amorphous ribbons listed in Table 1, or even comparable to those of some Zr-based bulk metallic glasses [37]. The liquidus temperatures (*T_l_*) of the Fe_88_Zr_8−x_Sm_x_B_4_ AA ribbons obtained from their melting curves, as displayed in the inset of Figure 1b, were approximately 1566 K for x = 2 and 1498 K for x = 4. Thus, the reduced glass transition temperature (*T_rg_*, defined as the ratio of *T_g_^onset^* to *T_l_*) [38] and the parameter *γ* (=*T_x_^onset^*/(*T_g_^onset^ + T_l_*)) [39] of the Fe_88_Zr_8−x_Sm_x_B_4_ AA ribbons were determined to be 0.453 and 0.378 for x = 2, and 0.478 and 0.390 for x = 4. Although the *T_rg_* of the Fe_88_Zr_8−x_Sm_x_B_4_ (x = 2, 4) AAs was not as large as that of the Fe_88_Zr_8_B_4_ alloy (*T_rg_* = 0.489) [31], their *γ* was much larger than that of the Fe_88_Zr_8_B_4_ alloy (*γ* = 0.350) due to the enlarged supercooled liquid region. Furthermore, the *γ* values of the Fe_88_Zr_8−x_Sm_x_B_4_ AAs were also much larger than that of other Fe-Zr-B-based AAs, as listed in Table 1, which made the Fe_88_Zr_8−x_Sm_x_B_4_ AAs exhibit a very large theoretical value of critical section thickness (*Z_c_*, ~1.96 mm for x = 2 and ~3.24 mm for x = 4) [39], even though the *T_rg_* of the Fe_88_Zr_8−x_Sm_x_B_4_ AAs was not high enough. As a result, the large values of ∆*T_x_*, *γ*, and *Z_c_* of the Fe_88_Zr_8−x_Sm_x_B_4_ AAs indicated that they showed a better thermal stability than other Fe-Zr-B-based amorphous ribbons and a sufficient GFA for the preparation of continuous amorphous ribbons.

It has been reported that the presence of nanocrystals promotes heterogeneous nucleation during crystallization and weakens the thermal stability of AAs [40], while the XRD and DSC results may not distinguish a small number of small-sized nanoparticles [41]. Therefore, in order to further confirm the amorphous structure of the two ribbons, the Fe_88_Zr_6_Sm_2_B_4_ amorphous ribbon with a lower GFA was employed for HREM observation. There were no obvious nanoparticles or a long-range order, but only 1~2 nm of a short-range order in the disordered matrix, as shown in Figure 1c, implying a fully amorphous state of the Fe_88_Zr_6_Sm_2_B_4_ ribbon.

### 3.3. Magnetic Properties of the Fe_88_Zr_8−x_Sm_x_B_4_ (x = 0, 2, 4) AAs

**Figure 2:**
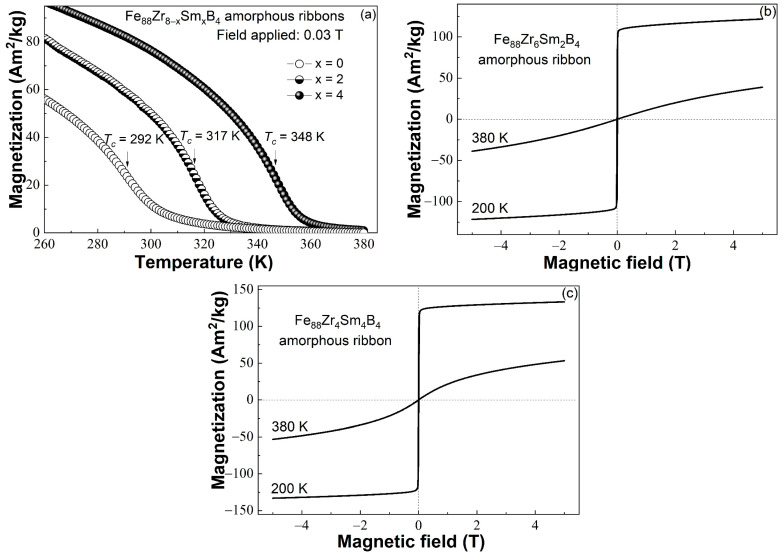
(**a**) The *M*-*T* curves of the Fe_88_Zr_8−x_Sm_x_B_4_ (x = 0, 2, 4) amorphous ribbons; the hysteresis loops of the (**b**) Fe_88_Zr_6_Sm_2_B_4_ and (**c**) Fe_88_Zr_4_Sm_4_B_4_ amorphous ribbons measured at 200 K and 380 K under 5 T.

The magnetization vs. temperature (*M*-*T*) curves of the Fe_88_Zr_8−x_Sm_x_B_4_ (x = 0, 2, 4) amorphous ribbons were measured from 200 K to 380 K under 0.03 T after a zero-field-cooling (ZFC) process from 300 K to 200 K, as shown in Figure 2a. Therefore, by taking one derivative of the *M*-*T* curves, the Curie temperature of the Fe_88_Zr_8−x_Sm_x_B_4_ AA samples was determined to be 292 K for x = 0, 317 K for x = 2, and 348 K for x = 4. The Sm addition dramatically improved the *T_c_* of the Fe_88_Zr_8_B_4_ AAs.

The hysteresis loops of the Fe_88_Zr_8−x_Sm_x_B_4_ (x = 2, 4) AAs measured under 5 T at 200 K and 380 K are illustrated in Figure 2b and Figure 2c, respectively. Both the samples were soft-magnetic at 200 K and paramagnetic at 380 K. The saturation magnetization (*M_s_*) reached 127 Am^2^/kg for the Fe_88_Zr_6_Sm_2_B_4_ amorphous sample and 139 Am^2^/kg for the Fe_88_Zr_4_Sm_4_B_4_ sample. The saturation magnetization of the Fe_88_Zr_8_B_4_ metallic glass (104 Am^2^/kg at 200 K [32]) was obviously improved by the Sm substitution, which indicated a better magnetocaloric performance of the Sm-doped samples because both the *M_s_* and the −Δ*S_m_^peak^* of the amorphous samples depended mainly on their ordering of magnetic moments.

### 3.4. Magnetocaloric Performance of the Fe_88_Zr_8−x_Sm_x_B_4_ (x = 2, 4) AAs

**Figure 3:**
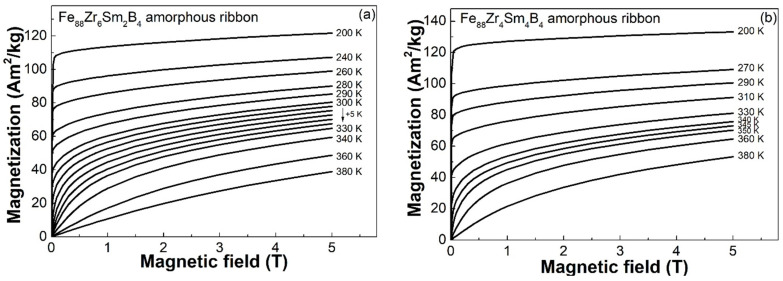
The isothermal magnetization curves of the (**a**) Fe_88_Zr_6_Sm_2_B_4_ and (**b**) Fe_88_Zr_4_Sm_4_B_4_ amorphous ribbons at various temperatures ranging from 200 K to 380 K under 5 T.

**Figure 4:**
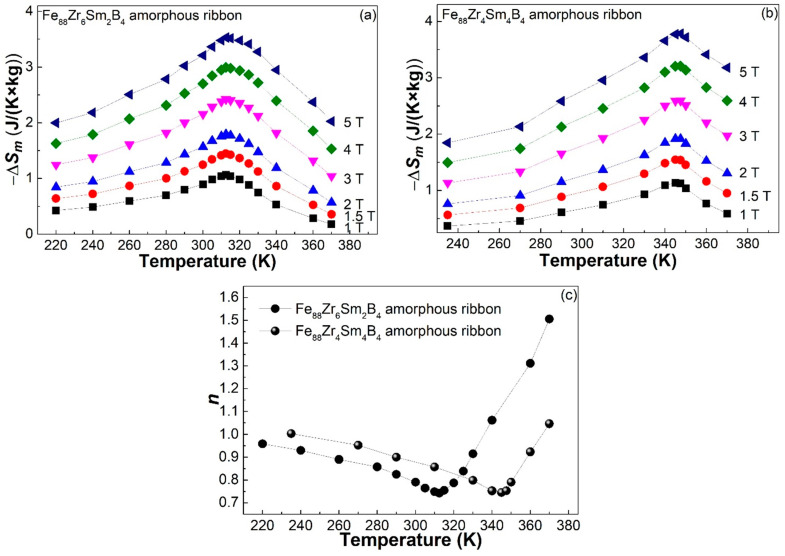
The −Δ*S_m_*-*T* curves of the (**a**) Fe_88_Zr_6_Sm_2_B_4_ and (**b**) Fe_88_Zr_4_Sm_4_B_4_ amorphous ribbons under various magnetic fields; (**c**) the temperature dependence of the exponent *n* for the Fe_88_Zr_8−x_Sm_x_B_4_ (x = 2, 4) amorphous ribbons.

Magnetic entropy changes in AAs are usually derived from their isothermal magnetization (*M*-*H*) curves measured at different temperatures. Figure 3 shows the *M*-*H* curves of the (a) Fe_88_Zr_6_Sm_2_B_4_ and (b) Fe_88_Zr_4_Sm_4_B_4_ amorphous samples. Thus, we calculated the −Δ*S_m_* at different temperatures (−Δ*S_m_*-*T* curve) of the Fe_88_Zr_8−x_Sm_x_B_4_ metallic glasses according to the Maxwell equation [42]. The −Δ*S_m_*-*T* curves of the Fe_88_Zr_8−x_Sm_x_B_4_ ribbons under different applied fields are depicted in Figure 4a,b. The −Δ*S_m_^peak^* of the two samples under 1 T, 1.5 T, 2 T, 3 T, 4 T, and 5 T are summarized in Table 2. As expected above, the −Δ*S_m_^peak^* under 5 T reached 3.53 J/(K × kg) near 317 K for the Fe_88_Zr_6_Sm_2_B_4_ ribbon and 3.79 J/(K × kg) near 348 K for the Fe_88_Zr_4_Sm_4_B_4_ ribbon, both of which were much higher than those of the Fe_88_Zr_8_B_4_ AA and other Fe-Zr-B-based amorphous ribbons [28,31,32,33]. As a result, the minor Sm substitution for Zr improved the magnetocaloric performance of the Fe_88_Zr_8_B_4_ metallic glass.

The magnetocaloric behaviors described by the *n*-*T* curves of the Fe_88_Zr_8−x_Sm_x_B_4_ amorphous samples are depicted in Figure 4c, where *n* is the slope of the linear fit for the ln(−Δ*S_m_*) vs. ln (*H*) plots. The shape of the *n*-*T* curves of the Fe_88_Zr_8−x_Sm_x_B_4_ metallic glass ribbons was similar to those of other amorphous alloys [32,43]. The minimum *n* value was ~0.74 at 312.5 K for the Fe_88_Zr_6_Sm_2_B_4_ ribbon and ~0.745 at 345 K for the Fe_88_Zr_4_Sm_4_B_4_ sample, both of which were close to the predicted value of fully amorphous alloys [43].

## 4. Discussion

### 4.1. Low-Temperature Magnetic Properties and Their Influence on the Magnetocaloric Properties of Sm-Doped Fe_88_Zr_8_B_4_ AA Amorphous Ribbons

It is argued that the introduction of RE atoms to iron-based amorphous alloys may give rise to an interaction between Fe and the RE atoms, and lead to the formation of randomly oriented anisotropic clusters. The coupling between these anisotropic clusters may result in spin-glass freezing behaviors and large hysteresis at low temperatures [44,45,46], which may be harmful to the magnetocaloric performance of RE-doped Fe-based AAs. Therefore, it is essential to study the low-temperature magnetic properties of Fe-based AAs containing RE elements.

Fortunately, the spin-glass freezing behaviors and hysteresis are usually negligible when the RE concentration is very low because the anisotropic clusters are hard to couple with each other due to the low density of these clusters [34,35]. Therefore, we selected the Fe_88_Zr_4_Sm_4_B_4_ AA that contained the highest Sm content in the present work to study its low-temperature magnetic properties and their possible effect on the magnetocaloric properties in more detail.

**Figure 5:**
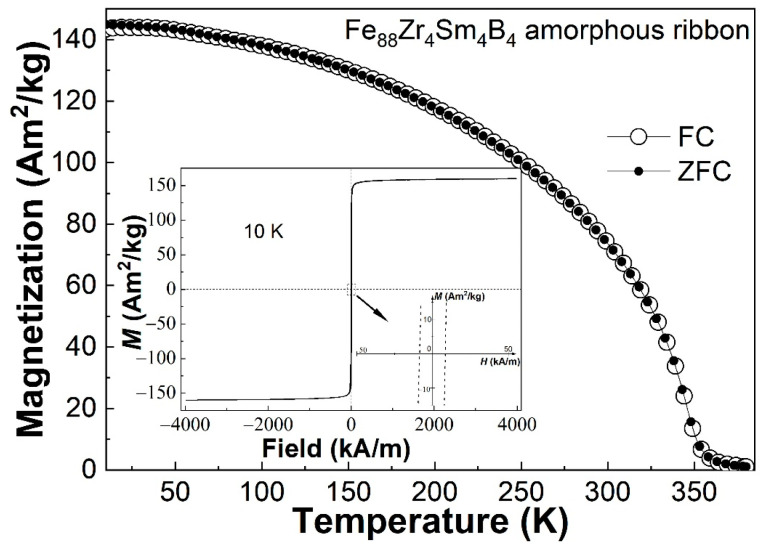
The ZFC and FC *M*-*T* curves of the Fe_88_Zr_4_Sm_4_B_4_ amorphous ribbon; the inset is the hysteresis loop of the Fe_88_Zr_4_Sm_4_B_4_ amorphous ribbon at 10 K.

Figure 5 shows the ZFC and field-cooling (FC, field applied: 0.03 T) *M*-*T* curves of the Fe_88_Zr_4_Sm_4_B_4_ AA sample. The ZFC *M*-*T* curve of the Fe_88_Zr_4_Sm_4_B_4_ AA ribbon almost overlapped the FC *M*-*T* curve, which implies that there was almost no spin-glass-like behavior in the Fe_88_Zr_4_Sm_4_B_4_ metallic glass. The hysteresis loop of the ribbon measured at 10 K is shown in the inset of Figure 5. Although the coercivity of the amorphous ribbon slightly increased compared to that of the Fe_88_Zr_8_B_4_ alloy (~4.89 kA/m [32]), its value was only 8.61 kA/m at 10 K; in other words, it was negligible even at 10 K. The invisible spin-glass-like behavior and negligible coercivity at low temperatures indicate that the low-concentration Sm substitution for Zr did not induce an obvious interaction between random anisotropic clusters, and thus, barely affected the magnetocaloric performance of the AA.

### 4.2. The Compositional Dependence of T_c_ in RE-Doped Fe_88_Zr_8_B_4_ AAs

**Figure 6:**
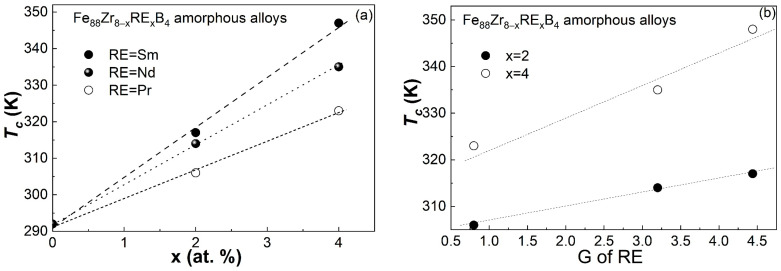
(**a**) The compositional dependence of *T_c_* for the Fe_88_Zr_8−x_RE_x_B_4_ (RE = Sm, Nd, Pr; x = 0, 2, 4) amorphous alloys; (**b**) the relationship between the *G* values of different RE types and their *T_c_* (solid circles represent x = 2 and hollow circles represent x = 4).

As is known, the tailorable Curie temperature is one of the major characteristics of AAs that are superior to intermetallic alloys. Therefore, it is vitally important to understand the mechanism for the compositional dependence of *T_c_* in these AAs. In the present work, the relationship between *T_c_* and the Sm concentration in the Fe_88_Zr_8−x_Sm_x_B_4_ AAs was observed and compared to the dependence of *T_c_* on the Nd and Pr content in the Fe_88_Zr_8−x_RE_x_B_4_ (RE = Nd, Pr) amorphous alloys. Figure 6a shows the *T_c_* of the Fe_88_Zr_8−x_RE_x_B_4_ (RE = Sm, Nd, Pr; x = 0, 2, 4) amorphous ribbons. The *T_c_* of the samples increased monotonously with the RE addition. The *T_c_* of the Sm-doped AAs increased faster than that of the Nd-doped AAs, and much faster than that of the Pr-doped AAs. Previous works that focused on the compositional dependence of *T_c_* in RE-TM-based AAs revealed that the *T_c_* of these AAs is influenced by the direct TM-TM interaction as well as the indirect RE-RE and RE-TM interactions [47]. Both the direct TM-TM interaction and indirect RE-RE interaction have a similar impact on *T_c_* in the RE-TM-based AAs; that is, *T_c_* changes linearly with the TM concentration or RE concentration. The relationship between the indirect RE-TM interaction and *T_c_* is more complicated and may exhibit an anti-parabolic shape. Fortunately, the negligible random magnetic anisotropy coupling mentioned above and the linear relationship between the *T_c_* and the RE content indicate that the Fe-RE interaction in Fe_88_Zr_8−x_RE_x_B_4_ AAs may be very weak or ignorable due to the low concentration of RE. On the other hand, as the Fe concentration in Fe_88_Zr_8−x_RE_x_B_4_ is fixed, the effect of the direct Fe-Fe interaction on the Curie temperature did not need to be considered in the present work. Therefore, the *T_c_* of the Fe_88_Zr_8−x_RE_x_B_4_ AAs depended mainly on the type of RE and its concentration. According to the Rudermann–Kittel–Kasuya–Yosida (RKKY) indirect interaction model, *T_c_* obeys a linear relationship with the de Gennes factor (*G*) of the RE in the AAs that contain only one rare earth element, as follows [48]:

$$T_{c}=\frac{2}{3k_{B}}I\left(0\right)G$$

where *k_B_* is the Boltzman constant and *I*(0) is the indirect exchange integral. From Figure 6a, one can find that *T_c_* increases linearly with the RE concentration when the kind of RE is fixed, which is related to the linear increase in the indirect exchange integral with the addition of RE. On the other hand, as the *G* value is 4.44 for Sm, 3.20 for Nd, and 0.80 for Pr, the *T_c_* of the Fe_88_Zr_8−x_Sm_x_B_4_ AAs is expected to be higher than the *T_c_* of the Fe_88_Zr_8−x_Nd_x_B_4_ AAs and much higher than the *T_c_* of the Fe_88_Zr_8−x_Pr_x_B_4_ AAs according to the equation when the RE concentration is fixed. Figure 6b shows the approximately linear dependence of *T_c_* on the *G* values of Sm, Nd, and Pr when x = 2 and x = 4 of the Fe_88_Zr_8−x_RE_x_B_4_, both of which demonstrated that the *T_c_* of the Fe_88_Zr_8−x_RE_x_B_4_ AAs is determined by the concentration and *G* value of the RE. The relationship between *T_c_* and the de Gennes factor also implies the effectiveness of the de Gennes factor in determining or adjusting the *T_c_* of RE-containing AAs.

### 4.3. Magnetocaloric Performance of RE-Doped Fe_88_Zr_8_B_4_ AAs

**Figure 7:**
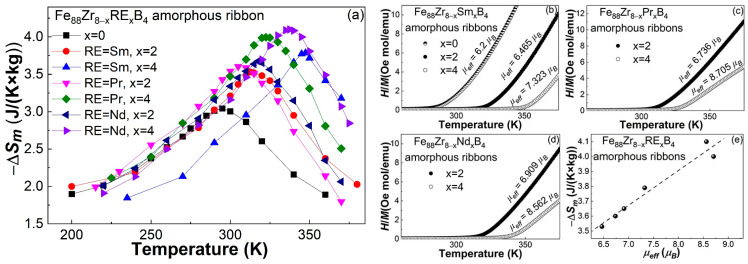
(**a**) The −Δ*S_m_*-*T* curves under 5 T of the Fe_88_Zr_8−x_RE_x_B_4_ (RE = Sm, Nd, Pr; x = 0, 2, 4) amorphous ribbons; the effective magnetic moment of the (**b**) Fe_88_Zr_8−x_Sm_x_B_4_ (x = 0, 2, 4), (**c**) Fe_88_Zr_8−x_Pr_x_B_4_ (x = 2, 4), and (**d**) Fe_88_Zr_8−x_Nd_x_B_4_ (x = 2, 4) amorphous alloys; and (**e**) the relationship between the −Δ*S_m_^peak^* and *µ_eff_* of the Fe_88_Zr_8−x_RE_x_B_4_ (RE = Sm, Nd, Pr; x = 2, 4) amorphous alloys.

Figure 7a shows the −Δ*S_m_*-*T* curves under 5 T of the Fe_88_Zr_8−x_Sm_x_B_4_ AA ribbons, and the Fe_88_Zr_8−x_Nd_x_B_4_ as well as the Fe_88_Zr_8−x_Pr_x_B_4_ AAs for comparison purposes. Apparently, the −Δ*S_m_^peak^* of Fe_88_Zr_8_B_4_ metallic glass was not only improved by a minor Sm substitution, but was also improved by Pr and Nd substitutions for Zr. Table 2 lists the *−*Δ*S_m_^peak^* under various magnetic fields of the Fe_88_Zr_8−x_RE_x_B_4_ (RE = Sm, Pr, Nd; x = 0, 2, 4) AAs. One can find that the improvement in the −Δ*S_m_^peak^* by the minor Sm addition was not as high as that resulting from the Pr and Nd additions at a fixed RE content; for example, the −Δ*S_m_^peak^* of the Fe_88_Zr_6_Sm_2_B_4_ amorphous ribbon at 5 T was ~3.53 J/(K × kg) at 317 K, which was lower than that of the Fe_88_Zr_6_Pr_2_B_4_ AA ribbon (~3.60 J/(K × kg) at 306 K [34]) and the Fe_88_Zr_6_Nd_2_B_4_ AA ribbon (~3.65 J/(K × kg) at 314 K [35]); the −Δ*S_m_^peak^* of the Fe_88_Zr_4_Sm_4_B_4_ amorphous ribbon at 5 T was ~3.79 J/(K × kg) at 348 K, which was lower than that of the Fe_88_Zr_4_Pr_4_B_4_ AA ribbon (~4.00 J/(K × kg) at 323 K [34]) and much lower than that of the Fe_88_Zr_6_Nd_2_B_4_ AA ribbon (~4.10 J/(K × kg) at 335 K [35]).

As the −Δ*S_m_* of the AAs depends mainly on the ordering of magnetic moments upon magnetization, an enhancement in −Δ*S_m_^peak^* by a small amount of RE as a substitution for Zr may be closely related to the extra magnetic moment produced by the introduction of RE atoms. The addition of different kinds or contents of RE elements may result in different −Δ*S_m_* improvements in the RE-doped amorphous alloys. As such, we attempted to calculate the effective magnetic moment (*µ_eff_*) of the Fe_88_Zr_8−x_RE_x_B_4_ AAs from their *M*-*T* curves based on the Curie–Weiss law and Langevin function [49]. As shown in Figure 7b–d, the *µ_eff_* of the Fe_88_Zr_8−x_RE_x_B_4_ amorphous ribbons was ~6.465 *µ_B_* for RE = Sm and x = 2, ~7.323 *µ_B_* for RE = Sm and x = 4, ~6.736 *µ_B_* for RE = Pr and x = 2, ~8.705 *µ_B_* for RE = Pr and x = 4, ~6.909 *µ_B_* for RE = Nd and x = 2, and ~8.652 *µ_B_* for RE = Nd and x = 4. Figure 7e shows the nearly linear relationship between the −Δ*S_m_^peak^* and the *µ_eff_* of the Fe_88_Zr_8−x_RE_x_B_4_ AAs, which indicates that the −Δ*S_m_^peak^* of the minor RE-doped Fe_88_Zr_8_B_4_ metallic glasses was closely related to their effective magnetic moment. Additionally, the *µ_eff_* of the Fe_88_Zr_8_B_4_ alloy was calculated to be ~6.2 *µ_B_*, as shown in Figure 7b. It was found that the addition of an RE element obviously enhanced the *µ_eff_* of the Fe_88_Zr_8_B_4_ alloy. The increased −Δ*S_m_^peak^* and enhanced *µ_eff_* suggest that an RE addition has a positive role in the improvement in the −Δ*S_m_^peak^* of Fe-based AAs.

## 5. Conclusions

Fe_88_Zr_8−x_Sm_x_B_4_ (x = 2, 4) amorphous samples with an average thickness of ~40 μm were successfully synthesized in this work. The GFA of the Fe_88_Zr_8−x_Sm_x_B_4_ (x = 2, 4) alloys was good enough for the 40-micrometer-thick amorphous ribbons. The effect of a Sm introduction on the magnetic and magnetocaloric performance of the Fe_88_Zr_8_B_4_ AA and the mechanism involved were studied in more detail by comparing with those of other RE additions. The main conclusions are as follows:

(a) The minor Sm-doped Fe_88_Zr_8_B_4_ AAs exhibited typical magnetocaloric behaviors of metallic glasses, and the invisible spin-glass-like behavior and negligible coercivity at low temperatures indicated that the minor Sm addition barely affected the magnetocaloric properties of the AAs.
(b) The *T_c_* of the Fe_88_Zr_8−x_Sm_x_B_4_ AAs increased faster than those of the Fe_88_Zr_8−x_Pr_x_B_4_ and Fe_88_Zr_8−x_Nd_x_B_4_ AAs. The nearly linear relationship between the *T_c_* and the de Gennes factor of Sm, Nd, and Pr when x = 2 and x = 4 demonstrated that the *T_c_* of the Fe_88_Zr_8−x_RE_x_B_4_ AAs was determined by the concentration and *G* value of the RE.
(c) Although the −Δ*S_m_^peak^* of the Fe_88_Zr_8_B_4_ AA was improved by the Sm substitution, the −Δ*S_m_^peak^* of the Fe_88_Zr_8−x_Sm_x_B_4_ was lower than that of the Fe_88_Zr_8−x_Pr_x_B_4_ and Fe_88_Zr_8−x_Nd_x_B_4_ amorphous ribbons when x = 2 and x = 4, respectively. The slightly lower −Δ*S_m_^peak^* of the Sm-doped AAs was closely related to their lower *µ_eff_* according to the roughly linear relationship between the −Δ*S_m_^peak^* and the *µ_eff_* of the Fe_88_Zr_8−x_RE_x_B_4_ AAs.

In brief, the mechanism of the change in the *T_c_* and −Δ*S_m_^peak^* of the Fe_88_Zr_8−x_RE_x_B_4_ AAs was revealed, and an RE addition had a positive effect on the magnetocaloric performance of Fe-based AAs. These results are expected to be helpful for achieving excellent magnetocaloric AAs with a high −Δ*S_m_^peak^* at a tailorable *T_c_* within the operating temperature interval of a domestic magnetic refrigerator.

## Figures and Tables

**Table 1:** The thermal parameters ∆*T_x_*, *T_rg_*, *γ*, and *Z_c_* of some Fe-Zr-B-based amorphous alloys.

Composition	*T_g_^onset^* (K)	*T_x_^onset^* (K)	*T_l_* (K)	∆*T_x_* (K)	*T_rg_*	*γ*	*Z_c_* (mm)	Refs.
Fe_88_Zr_6_Sm_2_B_4_	710	861	1566	151	0.453	0.378	1.96	Present work
Fe_88_Zr_4_Sm_4_B_4_	716	863	1498	147	0.478	0.390	3.24	
Fe_88_Zr_8_B_4_	787	840	1611	53	0.489	0.35	0.61	[31]
Fe_87_Co_1_Zr_8_B_4_	776	834	1580	58	0.491	0.354	0.72	
Fe_86_Co_2_Zr_8_B_4_	780	835	1574	55	0.496	0.355	0.75	
Fe_87_Zr_7_B_4_Co_2_	754	808	1570	54	0.48	0.348	0.56	
Fe_85_Co_3_Zr_5_B_4_Nb_3_	701	813	1495	112	0.469	0.370	1.41	
Fe_87_Zr_8_B_4_Sm_1_	828	859	1582	31	0.52	0.356	0.78	
Fe_86_Zr_8_B_4_Sm_2_	818	870	1580	52	0.52	0.363	1.05	
Fe_85_Zr_8_B_4_Sm_3_	828	883	1578	55	0.53	0.367	1.24	
Fe_88_Zr_6_B_4_Ti_2_	752	812	1539	60	0.489	0.354	0.72	[32]
Fe_88_Zr_6_Pr_2_B_4_	757	866	1509	109	0.482	0.377	1.88	[34]
Fe_88_Zr_4_Pr_4_B_4_	768	865	1527	97	0.503	0.372	1.53	

**Table 2:** *T_c_* and −Δ*S_m_^peak^* of the Fe_88_Zr_8−x_RE_x_B_4_ (RE = Sm, Nd, Pr; x = 0, 2, 4) amorphous alloys.

Composition	−Δ*S_m_^peak^* (J/(kg × K))						*T_c_* (K)	Refs.
	1 T	1.5 T	2 T	3 T	4 T	5 T		
Fe_88_Zr_6_Sm_2_B_4_ ^a^	1.07	1.45	1.79	2.42	3.00	3.53	317	Present work
Fe_88_Zr_4_Sm_4_B_4_ ^a^	1.14	1.54	1.92	2.59	3.21	3.79	348	
Fe_88_Zr_8_B_4_	0.87	1.20	1.50	2.06	2.57	3.04	292	[32]
Fe_88_Zr_6_Nd_2_B_4_	1.09	1.49	1.84	2.50	3.09	3.65	314	[35]
Fe_88_Zr_4_Nd_4_B_4_	1.20	1.65	2.05	2.79	3.47	4.10	335	
Fe_88_Zr_6_Pr_2_B_4_	1.07	1.47	1.82	2.46	3.05	3.60	306	[34]
Fe_88_Zr_4_Pr_4_B_4_	1.20	1.63	2.02	2.74	3.39	4.00	323	

^a^ These results were obtained for the first time.

## Data Availability

Data are contained within the article.
